# Room temperature manipulation of long lifetime spins in metallic-like carbon nanospheres

**DOI:** 10.1038/ncomms12232

**Published:** 2016-07-18

**Authors:** Bálint Náfrádi, Mohammad Choucair, Klaus-Peter Dinse, László Forró

**Affiliations:** 1 Laboratory of Physics of Complex Matter (LPMC), Ecole Polytechnique Fédérale de Lausanne, 1015 Lausanne, Switzerland; 2 School of Chemistry, University of Sydney, Sydney, New South Wales 2006, Australia; 3 Institut für Experimentalphysik, Freie Universität Berlin, Arnimallee 14, 14195 Berlin, Germany

## Abstract

The time-window for processing electron spin information (spintronics) in solid-state quantum electronic devices is determined by the spin–lattice and spin–spin relaxation times of electrons. Minimizing the effects of spin–orbit coupling and the local magnetic contributions of neighbouring atoms on spin–lattice and spin–spin relaxation times at room temperature remain substantial challenges to practical spintronics. Here we report conduction electron spin–lattice and spin–spin relaxation times of 175 ns at 300 K in 37±7 nm carbon spheres, which is remarkably long for any conducting solid-state material of comparable size. Following the observation of spin polarization by electron spin resonance, we control the quantum state of the electron spin by applying short bursts of an oscillating magnetic field and observe coherent oscillations of the spin state. These results demonstrate the feasibility of operating electron spins in conducting carbon nanospheres as quantum bits at room temperature.

## Abstract (web summary)

Electronic decoherence due to spin-orbit and magnetic interactions limits the application of spintronic nanosystems in quantum information processing. Here, the authors report notably long spin-lattice and spin-spin relaxation times of 175 ns at room temperature in carbon nanospheres.

## Graphical abstract

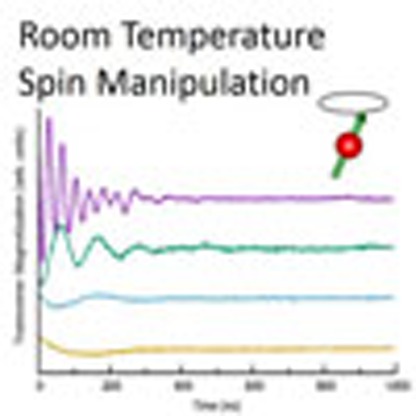

Electron spin states are an attractive realization of a quantum bit (qubit) as they can undergo a transition between the spin-up and spin-down quantum states^1^. The most commonly used technique for manipulating electron spin is electron spin resonance (ESR)^2^. ESR is the physical process, whereby electron spins are polarized in an external magnetic field **B**_0_ and rotated by an oscillating magnetic field **B**_1_ (perpendicularly to **B**_0_, of frequency *f*), which is resonant with the spin precession frequency in an external magnetic field *f*=*gμ*_B_B_0_/*h* (*μ*_B_ is the Bohr magneton and *g* the electron spin *g*-factor, *h* is the Planck's constant). ESR is result of the coherence of the precession of electrons over the spin–lattice and spin–spin relaxation times, *T*_1_ and *T*_2_, respectively^3^. *T*_1_ is characterized by a number of spin–lattice relaxation process that depend on the spin–orbit coupling to connect the spin of an electron with the lattice vibrational spectrum of the solid. The second important relaxation time *T*_2_ is set by the probability of spin–spin relaxation. *T*_2_ is concerned with the local magnetic field contribution by one magnetic atom on others and represents the phase coherence of a set of spins. The dominant relaxation time is the shorter of *T*_1_ and *T*_2_. In magnetically homogenous itinerant systems (for example, metals), the condition *T*_1_=*T*_2_ is often met and represents the longest period of time that in-phase precessing electron spins and magnetization can propagate as a uniform mode^4^.

Electron spin states therefore need to be robust against decoherence. The feasibility of applications involving classical or quantum information processing is hence critically dependent on *T*_1_ and *T*_2_ relaxation times^5^,^6^,^7^,^8^,^9^,^10^,^11^,^12^. The prerequisite for *T*_1_ and *T*_2_ relaxation times is ∼100 ns, as this is the state of the art lower-bound for signal processing times in quantum electronic devices^13^,^14^.

Advances in the fields of inorganic^15^,^16^ and molecular^17^,^18^ quantum dots have made the electron spin system promising for practical application in spintronic and quantum information processing. Current research activities on spin-based qubits are divided into two distinct research directions with limited overlap and are based on the materials employed: inorganic materials, usually semiconducting structures forming quantum dots, and carbon-based molecules. The qubits utilized in these materials are also different: inorganic materials containing localized spins of doped ions or conduction electron spin confined on quantum dots, whereas organic materials utilize localized paramagnetic spins confined on molecules.

Due to the radical differences in the materials and the electron systems employed for spin qubit manipulation, the major challenges to realising devices are also distinct. For solid-state inorganic materials the strong spin–orbit interaction enforced by heavy nuclei in materials like semiconductors^10^,^11^,^19^ and metals^12^,^20^,^21^ strongly requires the pursuit of structural perfection and spin manipulation at low temperatures (40 mK to 100 K). This has resulted in well-defined materials that currently set the benchmark for long *T*_1_ relaxation times exceeding seconds^9^. Unfortunately, at room temperatures a number of intrinsic spin–lattice relaxation processes and phonon modes induce spin decoherence and diminish *T*_1_ and *T*_2_ (ref. 22). Consequently, for metal nanoparticles^21^ *T*_1_ and *T*_2_ shortens to 10–40 ps and for bulk semiconductors^23^,^24^ to 1–4 ns.

Molecular qubits can be produced on an industrial scale and are readily processed^18^. These spin-bearing organic molecules often have low spin–orbit coupling, possess many non-degenerate spin transitions, and can be chemically modified. These attributes have led to the potential of molecular compounds for both spintronics and quantum information processing. However, the dipole–dipole interactions between molecules and the hyperfine interaction of the molecule requires diluting the spin containing molecules and isotope engineering of the host system to reduce magnetic inhomogeneity. It also needs low temperatures (<100 K) for spin manipulation to dampen molecular vibrations that cause spin decoherence.

Attempts to combine the advantages of inorganic and molecular qubit materials have led to electronically insulating systems like nitrogen-vacancy (N-V) centre nanodiamonds^25^ and N@C_60_ (refs 14, 26). Long microsecond *T*_1_ and *T*_2_ of localized electrons in these materials can be achieved and manipulated at ambient conditions. Magnetic inhomogeneity in these materials is accounted for by isotopic engineering constituent ^13^C and ^14,15^N nuclei, however, the localized electron spins themselves generate fluctuating magnetic fields, posing limitations on the qubit density. This limitation is specific to localized electron systems and it is absent for conduction electrons where the motional narrowing process negates this effect^27^.

Carbon nanotube-based quantum dots have been prepared with an electron spin dephasing time of ∼3 ns at millikelvin temperature^28^. The shortening of *T*_2_ in these carbon nanotubes is attributed to an increase in the spin–orbit coupling induced by the high curvature of the carbon sheets^29^. Graphene on the other hand is comprised entirely from a single-atom thick crystalline layer of carbon, is flat (or at best not curved), and conducting^30^,^31^. Experiments on single- and few-layer graphene have demonstrated *T*_1_=*T*_2_ of 1–4 ns at room temperature^32^. However, this value is still far below expectations for practical use and possibly limited by physisorption and chemisorption of molecular oxygen or other species onto the unprotected carbon surface^33^. Non-carbon-based materials have also been developed, including heterometallic molecular nanomagnets comprising of isotopically engineered and diamagnetically diluted Cr_7_M (M=Ni, Mn, Zn) that demonstrate the prerequisite for quantum processing applications at low temperature (5 K) (ref. 34).

These possible solutions demonstrate that there are clear and established trade-offs to be considered regarding the feasibility of applying a qubit material system: a conducting material of light atomic weight constituents that meets the prerequisite *T*_1_ and *T*_2_ at room temperature would permit real spintronics and quantum processing applications. Such a material would combine the best aspects of both inorganic and molecular spin qubit schemes.

Here we report the observation of *T*_1_=*T*_2_=175 ns at room temperature (300 K) in conducting metallic-like carbon nanospheres via ESR. Furthermore, we could coherently control the quantum state of the electron spin confined on the carbon nanospheres at 300 K. This remarkable result can be put in context by comparing it with other promising systems, which could carry the quantum bit: N@C_60_, N-V centre in nanodiamond or P-doped Si (Fig. 1). In the carbon nanospheres, the electron spins are highly delocalized and polarizable (Pauli-paramagnetic) over the volume of the nanosphere, rather than highly confined to a small path on the order of the electron orbit, as would be the case for a localized electron system. This has the advantages that there is no need for isotopic engineering of the carbon nuclei to suppress magnetic inhomogeneity, and an absence of fluctuations in local magnetic fields of localized spins. The material thus allows a higher density packing of qubits to be, in principle, achieved over other promising qubits.

## Results

**Figure 2:**
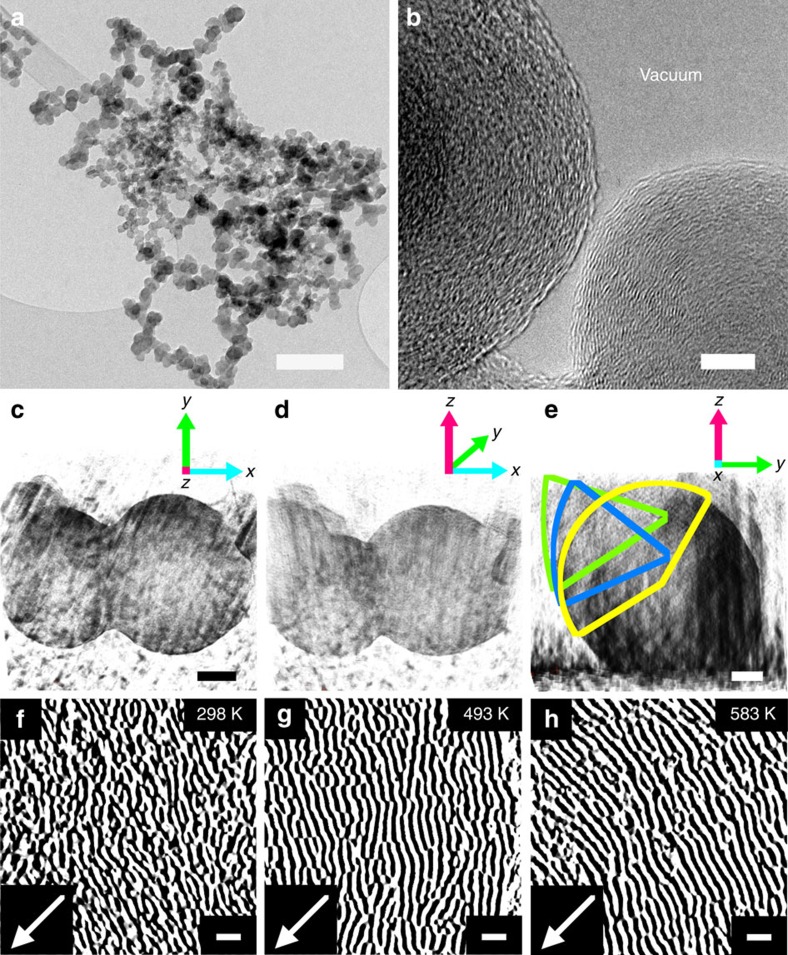
Carbon nanosphere structure and size. (**a**) TEM image of carbon nanospheres on a carbon support. (**b**) High-resolution TEM of discrete carbon nanospheres. (**c**–**e**) TEM three-dimensional tomography reconstruction of the carbon nanospheres. The particles are spherical and also distort slightly to an ellipsoidal shape, with coalescence. Spheres appear transparent due to the high image contrast with the sputtered gold substrate, small sphere size and extremely thin sphere layers. (**f**–**h**) *In situ* variable temperature high-resolution TEM images of the non-crystalline carbon nanosphere structure within regions of various spheres. The spheres remain non-crystalline upon heating to 583 K. A high contrast is applied to the images to allow the graphite planes to be distinguished with a black outline. Arrows indicate direction towards the centre of the sphere. Scale bars, 200 nm (**a**), 5 nm (**b**), 10 nm (**c**), were omitted for clarity (**d**), 5 nm (**e**), 1 nm (**f**–**h**).

The structural and chemical properties of the carbon nanospheres are inherently key to the observation of the long *T*_1_=*T*_2_. Transmission electron microscopy (TEM) images show the extensive formation of spherical carbon spanning micron scales (Fig. 2a and Supplementary Fig. 1). The as-prepared carbon nanospheres are a conglomeration of spherical bodies and after sonication in suspension they could appear as individual particles (Fig. 2b). In comparison with other nanoparticle quantum dots used as qubits, for example, Mn-doped PbS (ref. 35), the carbon nanospheres are relatively uniform with a size distribution of 37±7 nm estimated from TEM images (Supplementary Fig. 2).

By means of TEM tomography, a number of carbon nanospheres are also observed to have an asymmetric shape, which also results from the formation of joint graphitic layers of contacting particles (Fig. 2c–e), with the accretion of layers between nanospheres generally forming within a region of ca. 5 nm of the outer layers (Supplementary Fig. 3). High-resolution TEM revealed the short graphitic fragments that comprise the carbon nanospheres are graphitic fragments that follow the curvature of a sphere, creating many open edges (Fig. 2f–h). The individual graphitic fragments in the carbon nanospheres are not curved and do not resemble the curvature in nanotubes or fullerenes. Rather, the fragments exhibit an intricate array of interplanar bonding all the way to the centre of the nanosphere even when heated to temperatures of 583 K. The carbon nanospheres are not hollow and show a continuation of the closed cage structure towards the centre.

X-ray photoelectron spectroscopy (XPS), thermogravimetric analysis (TGA), and Raman experiments were also performed and are found in Supplementary Figs 4–9 and Supplementary Notes 1–3. XPS indicated that the chemical structure is predominately conducting graphitic carbon (90.2 wt%) containing surface bound oxygen (9.8 wt%), and there was no inclusion of metals and other heavy atoms. TGA experiments confirmed the carbon nanosphere material did not contain residual precursor polyaromatic hydrocarbons and remained chemically and thermally stable even up to temperatures of 883 K. Valence band XPS revealed the presence of non-bonding *π* and *σ* orbitals as a result of fragmented sheets that contained carbon arranged in a distorted hexagonal network.

**Figure 3:**
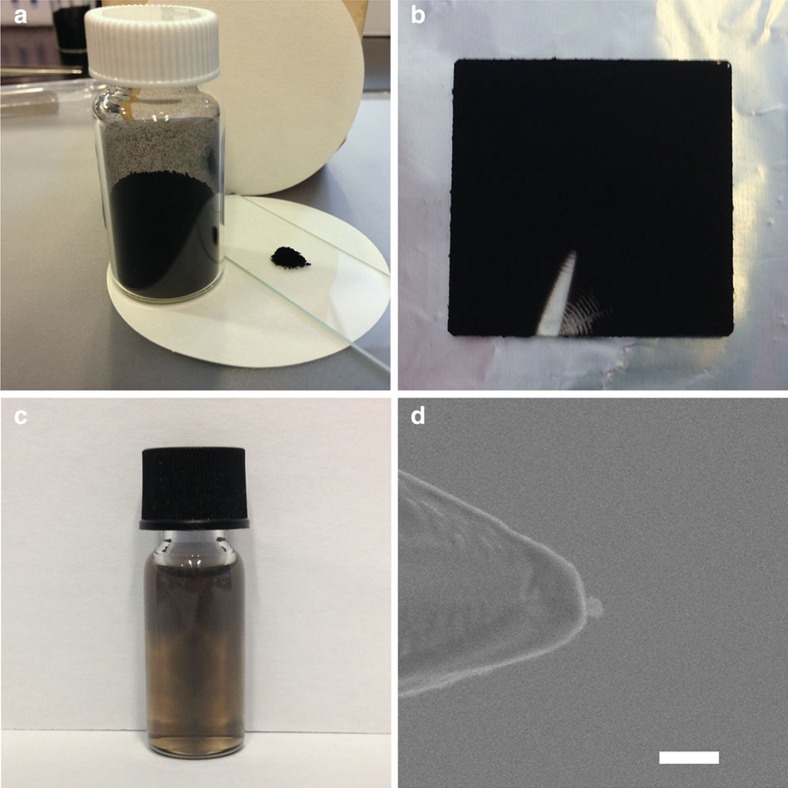
Sample processing of carbon nanospheres. (**a**) A 300 mg of carbon nanospheres collected as a solid powder in a sample tube and 10 mg on a glass slide, stable in air. (**b**) Carbon nanospheres directly deposited during synthesis onto a 2.5 × 2.5 cm quartz slide. (**c**) A small amount of carbon nanospheres dispersed in 2 ml of ethanol by sonication. (**d**) SEM image of an individual ∼50 nm carbon nanosphere physically positioned on a Si substrate using a 200-nm tungsten manipulator tip. Scale bar, 200 nm (**d**).

The carbon nanospheres are easily synthesized^36^ and readily processable (Fig. 3), yielding a homogenous material that is structurally highly non-crystalline (Fig. 2). Thus the carbon nanospheres can be reliably employed for spintronics applications with minimal processing and without the need for fabricating a well-defined crystal structure to achieve long *T*_1_ and *T*_2_. Furthermore, in a conducting carbon nanosphere qubit system, the rich chemistry of carbon can, in future experiments, allow for a myriad of non-covalent and covalent interactions to connect the nanospheres to conducting electronic device surfaces^37^,^38^. Finally, the carbon nanospheres are of a size that can be isolated on a surface from the ‘top-down' using micromanipulator probe tips (Fig. 3d), and this in future experiments can allow for building qubit ensembles^39^.

**Figure 4:**
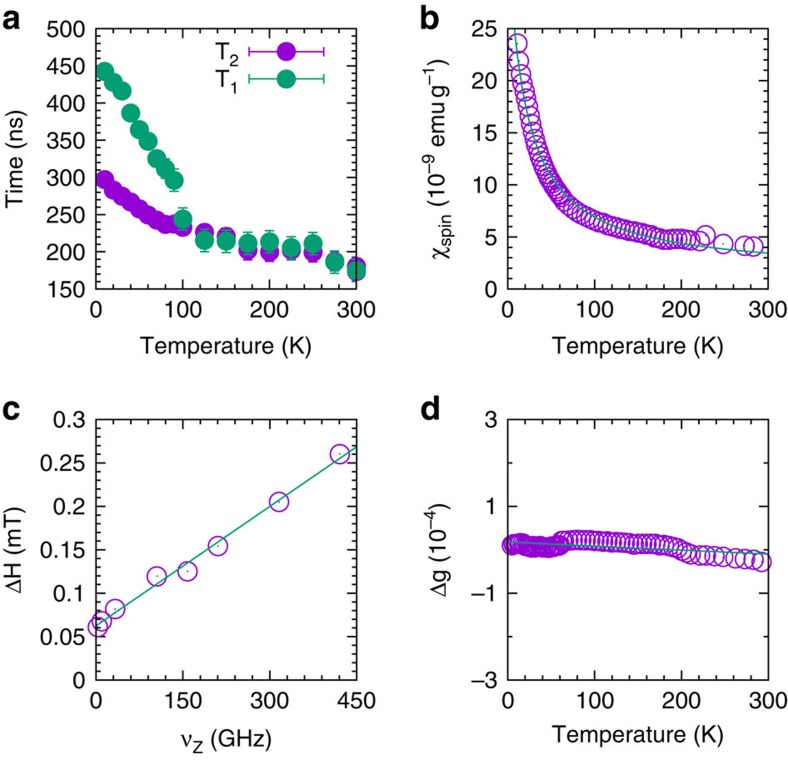
Characterization of the spin system of the carbon spheres by ESR. (**a**)Temperature dependence of *T*_1_ and *T*_2_ at *ν*_Z_=9.5 GHz. (**b**)Temperature dependence of the spin susceptibility, *χ*_spin_, measured by ESR at *ν*_Z_=9.4 GHz with an overlaying Curie−Weiss line, characteristic to small paramagnetic particles. (**c**) The ESR linewidth is plotted as a function of the Zeeman energy, *E*_Z_=*hν*_Z_ measured by a multi-frequency ESR at 300 K. The linear fit (straight solid line) using equation (1), with *T*_1_=*T*_2_=175 ns gives *δ*=1 meV. (**d**) The temperature-independent *g*-factor shift Δ*g* relative to the free electron *g*-value, in good agreement with a material exhibiting very weak spin−orbit coupling. Error bars represent the confidence interval of least square fits to the spectra.

Here we develop in more detail our key observations obtained by ESR. At 300 K and at 9.4 GHz frequency the continuous-wave ESR line width (peak-to-peak) is Δ*H*=0.056 mT (inset Fig. 4a and Supplementary Fig. 10) and the *g*-value is 2.00225 (inset Fig. 4b). This is a remarkably narrow conduction-electron spin ESR line testifying the long spin-relaxation times. The observed spectra had, to a high precision, homogeneously broadened Lorentzian line shapes and the deviation from the Lorentzian line shape in the entire spectra was <5%, (see Supplementary Fig. 10 as an example), which reveals the itinerant nature of the spins. The observed linewidth determined by continuous-wave ESR is identical within the experimental error with the *T*_2_-derived Lorentzian width. Note that the size distribution of the carbon nanospheres has a negligible effect on the linewidth at 9.4 GHz because of the motional narrowing of conduction electrons (Supplementary Fig. 11). The hyperfine ESR lines of ^13^C were also absent (which are readily observable for localized spins^14^,^25^,^26^) due to motional narrowing of conduction electrons^27^. The *g*-factor is characteristic to conduction electrons of carbon, and it does not originate from metallic inclusions (in agreement with our chemical analysis) or from localized paramagnetic ‘dangling' bonds of carbon (commonly with *g*=2.00282) (ref. 40).

In addition to the continuous-wave ESR experiment, where detection and spin rotation occur at the same time, we extended our experiments to probe the spin relaxation dynamics of *T*_1_ and *T*_2_ independently using pulsed ESR (Fig. 4a). At 9.5 GHz frequency and 300 K, with good approximation we found that the intrinsic *T*_1_=*T*_2_=175 ns. Pulsed ESR therefore simultaneously validated our continuous-wave ESR results and verified that the line-shape obtained by continuous-wave ESR was indeed homogenous as expected for itinerant electrons.

The temperature-dependent properties of the ESR spectra (Fig. 4 and see also Supplementary Fig. 12) support that conduction electrons are confined within the carbon nanospheres. The *g*-factor was temperature independent (Fig. 4d), which is in good agreement with general observations in metals with weak spin–orbit coupling and in graphitic nanoparticles^41^. The resulting spin susceptibility is temperature dependent, following a Curie–Weiss dependence, as one may expect for nanoparticles of metals (Fig. 4b)^21^,^42^.

Multi-frequency ESR in the 4–420 GHz frequency and 2–300 K temperature range also confirmed that conduction electrons are confined within the carbon nanoparticles (Fig. 4 and Supplementary Fig. 12)^43^,^44^. The ESR linewidth revealed a linear increase with increasing magnetic field at 300 K (Fig. 4c and see also Supplementary Fig. 12). Note that in the case of bulk metals, Δ*H* is solely determined by spin–orbit coupling thus it is independent of the magnetic field^21^,^45^. However, the behaviour observed when the carbon nanospheres experience a variation in external magnetic field is characteristic to conduction electrons enclosed in nanoparticles where *T*_1_ and *T*_2_ are determined by both the spin–orbit interaction and electron confinement^21^. This broadening of Δ*H* for itinerant electrons confined on small particles follows^46^:


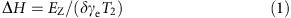


where *E*_Z_*=hν*_Z_ is the Zeeman energy, *δ* is the average electronic energy level spacing and *γ*_e_ is the electron gyromagnetic ratio^46^. Using the measured *T*_1_=*T*_2_=175 ns, we can extract a *δ*=1 meV for the average electronic energy level spacing (linear line of best fit in Fig. 4c). From this value one can calculate back the size of the carbon spheres by following Kubo's calculations for a small, almost spherical, metallic particle^42^:


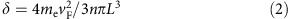


where *m*_e_ is the free electron mass, *v*_F_=10^6^ ms^−1^ is a typical Fermi velocity for graphene, and *n*=2.3 g cm^−3^ is the atomic density of the carbon nanospheres. These values yield an effective linear particle size of *L*=40 nm. This particle size is in good agreement with that obtained from TEM images. Furthermore, the increase in *T*_1_ and *T*_2_ as the temperature is decreased (Fig. 4a and see also Supplementary Fig. 12) is characteristic to metals^21^,^41^,^45^, where electron–phonon scattering due to spin–orbit interaction is responsible for the temperature-induced shortening of *T*_1_ and *T*_2_ (refs 42, 45, 46).

As the temperature was decreased, *T*_2_ reached 300 ns at 4 K while *T*_1_ reached 450 ns (Fig. 4a). There is a deviation from the *T*_1_*=T*_2_ dependence below ∼100 K. During the delineation of *T*_*1*_ and *T*_2_ below ∼100 K, *T*_1_ and *T*_2_ nevertheless continue to increase at different rates. The electron spin dynamics of *T*_1_ is directly related to phonon dampening in disordered graphitic sheets^27^,^41^ and the existence of Wallis-type^47^ local phonon modes. Future studies have to show if further localization at specific defect sites occurs at very low temperatures. As the temperature is increased, the calculations of Andersson *et al*.^41^ indicate that the observed shortening of *T*_1_ and *T*_2_ is caused by the scattering of conduction electrons by the potentials of peripheral atoms having edge-inherited electronic and lattice dynamical features and the excitation of low-energy phonons.

From our structural, chemical and electronic characterization we propose that the non-bonding orbitals associated to the structural imperfections induce conduction electrons to the system and significantly enhance the electron density of states. This is in agreement with theoretical works^41^,^48^ that predict the presence of an additional band superimposed upon the bonding *π* and the anti-bonding π* bands around the Fermi energy in nanometre size disordered graphitic fragments, and our observations of the changes in the *p*_z_ wave functions in the *p*–*π* band evolutions with temperature near the Fermi energy level (Supplementary Fig. 5).

The carbon nanospheres also contain covalently bonded oxygen that contributes to the disorder within the graphitic lattices (Fig. 3f–h; Supplementary Table 1; Supplementary Fig. 9). We note that the removal of oxygen groups by thermal decomposition introduces non-bonding *π* and *σ* orbitals (Supplementary Figs 4 and 5). In future experiments, greater robustness against spin decoherence may be achieved by the removal of adatoms to enhance the electronic density of states^33^.

Although the carbon nanospheres are highly defective, the intrinsically weak spin−orbit interaction of carbon has allowed for long *T*_1_ and *T*_2_ to persist even at 300 K. In nanotubes and fullerenes, an increase in spin−orbit coupling due to graphene sheet curvature may contribute to the shortening of *T*_1_ and *T*_2_ (refs 28, 29), however the individual graphitic flakes in the carbon nanospheres are not curved. The *T*_1_=*T*_2_ in the carbon nanospheres is remarkably a two orders of magnitude enhancement over that found in conducting crystalline graphene^32^. We attribute part of this increase in *T*_1_ and *T*_2_ to quantum confinement effects readily observed in conducting nanostructures^9^,^11^,^12^,^21^.

**Figure 5:**
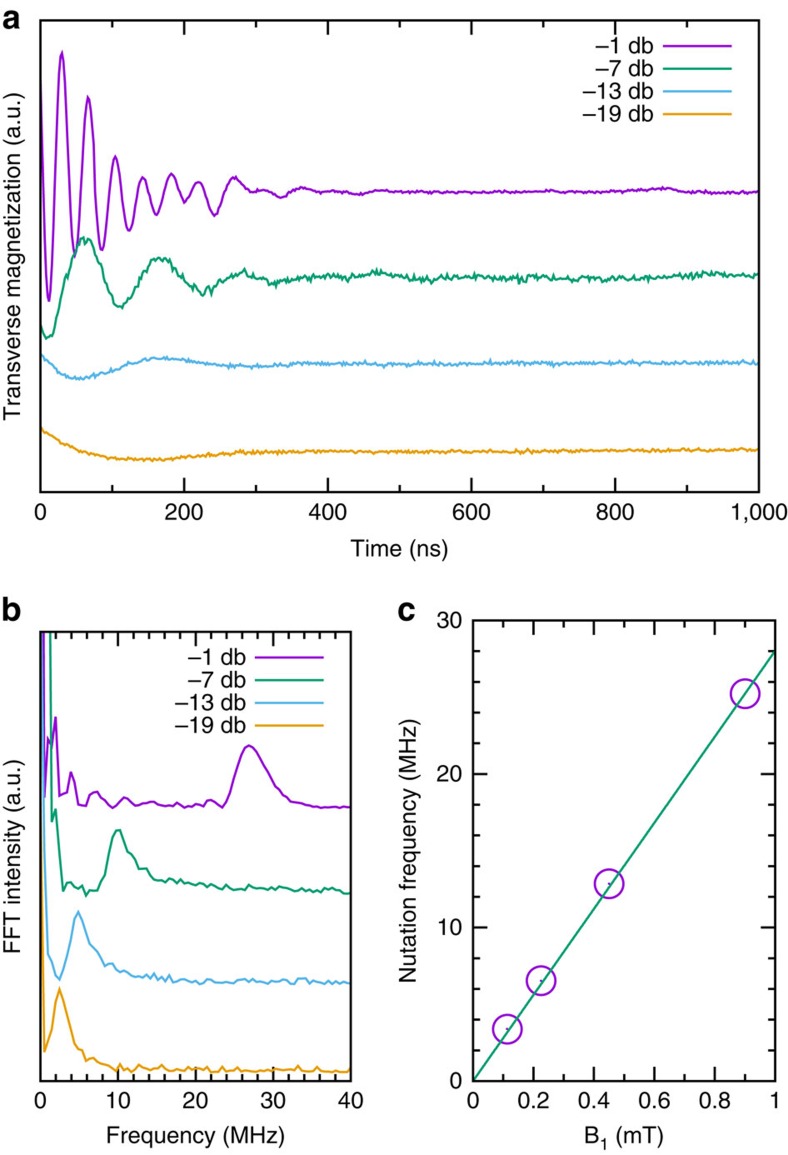
Spin control over electron qubits confined to carbon nanospheres at 300 K. (**a**) Rabi oscillations of the electron qubits at 300 K and *B*_0_=337 mT for different microwave powers. (**b**) Fourier transform of the Rabi oscillations, with the signal shifted vertically for clarity. (**c**) Rabi frequency is proportional with the square root of the power. The value 25 MHz observed for the maximum power is consistent with the previously determined maximum *B*_1_ field in the dielectric cavity of 0.9 mT, indicating the presence of an effective spin *S*=1/2.

Following our observations of magnetically induced spin polarization, we coherently rotated the electron spin at 300 K by applying microwave power bursts of increasing duration and with variable power (Fig. 5a). We observed that the magnetization of the carbon nanospheres oscillates periodically with pulse duration, the oscillation frequency being proportional to the square root of the microwave power. This oscillation indicates that we have performed deliberate and coherent electron spin rotations (driving of electron spins between two Zeeman-split energy levels), or Rabi oscillations. This is the sign that one can manipulate spins both for spintronics and quantum information processing (see also Supplementary Note 4)^1^,^49^. Fourier-transformed Rabi oscillation signals show a single-component characteristic to electron spin-1/2 qubit (Fig. 5b). A key characteristic of the Rabi process is a linear dependence of the Rabi frequency on the microwave field strength **B**_1_ (*f*_Rabi_=*gμ*_B_**B**_1_/*h*). We verify this by extracting the Rabi frequency from a fit of the Fourier-transformed signal of Fig. 5b with a Gaussian line, which gives the expected linear behaviour that is proportional to **B**_1_ (Fig. 5c). This demonstrated the capacity to rotate the spin qubits on the carbon nanospheres arbitrarily to any point on the Bloch sphere^3^ at temperatures as high as 300 K. Rabi oscillations can be observed for ∼400 ns, consistent with decoherence by *T*_2_. The increase in linewidth of the Fourier-transformed oscillations with microwave power (Fig. 5b) is caused by microwave field inhomogeneity.

## Discussion

We have demonstrated that a long itinerant electron spin lifetime in a magnetically homogenous conducting material can be achieved at room temperature. Through the controlled polarization of coherent electron spins in these carbon nanospheres, we have showed that this electron spin lifetime exceeds the prerequisite for applications in spintronics and quantum information processing. This is possible through electron confinement to nanometre-sized, non-crystalline yet metallic-like carbon spheres. This work effectively bridges the disparate research directions in the fields of inorganic and molecular materials for electron spin qubits and has broad applicability: spin qubits can now be manipulated at room temperature without the need for isotopically engineering a host material, diluting the spin-carrying molecule, cryogenic temperatures, the preparation of well-defined crystal structures, or the use of metals. The facile preparation of a carbon material using common laboratory reagents, combined with the use of well-established electron spin manipulation measurements at room temperature, effectively reduces many of the technological barriers to realising practical quantum computing and spintronics using solid-state materials.

The spin-manipulation experiments described here were performed on a large number of carbon nanospheres. Although the material can be readily chemically processed, it is prepared in a form suitable for device processing: we have demonstrated that the conducting nanospheres can be isolated on a silicon surface by physically manipulating individual nanospheres. In principal, this may provide an initial avenue to high-density qubit arrays of nanospheres that are integrated onto existing silicon technologies or thin-film-based electronics.

## Methods

### Sample preparation

The preparation of the carbon nanospheres is described in detail elsewhere^36^ and can be summarized as the soot product resulting from the partial combustion of naphthalene in air, which is collected and heated at 473 K under dynamic vacuum for 72 h. Approximately 100 mg was prepared for all experimental procedures.

### Transmission electron microscopy

Samples were analysed using a field emission JEOL3000F operated at 300 kV. Particle-size distribution and topography image analysis was performed using freely available ImageJ 1.48v software (http://imagej.nih.gov/ij). Energy-filtered tomography images were obtained using a JEOL JEM 2200FS Field Emission Microscope operated at 200 kV using a high-tilt holder, with an in-column omega filter and objective aperture applied. JEOL recorder software v2.48.1.1 was used to collect the topography images. JEOL Composer and JEOL Visualizer-kai programs were used to reconstruct the tomography images.

### Scanning electron microscopy

Scanning electron microscopy was performed using a Zeiss Ultra Plus.

### X-ray photoelectron spectroscopy

Measurements were conducted using an ESCALAB250Xi instrument manufactured by Thermo Scientific, UK. The background vacuum was better than 2 × 10^−9^ mbar. A monochromated Al *K*_*α*_ (energy *hν*=1,486.68 eV) was used with a spot size of 500 μm. The fine carbon powder was manually pressed onto indium foil for analysis or pressed into a disc prior to *in situ* heating experiments. Curve fitting was performed using the Scienta ESCA300 data-system software. Binding energy reference was C 1*s*=285.0 eV for adventitious carbon.

### Raman spectroscopy

Raman spectroscopy was performed using Argon 514 nm excitation laser on a Renishaw Raman inVia Reflex with a notch and edge filter cutoff of 100 cm^−1^.

### Modulated thermogravimetric analysis

Modulated thermogravimetric analysis measurements were obtained using a TA HiRes Discovery TGA in modulated TGA mode with a heating profile of 2 °C min^−1^ under the flow of 20 ml min^−1^ high-purity N_2_ with sinusoidal temperature amplitude of 4 °C and period of 200 s. A 100-μl alumina pan was used. Evolved gas analysis (TGA GC–MS) was performed using a Perkin Elmer Thermogravimetric Analyzer Pyris 1 coupled to a Perkin Elmer Gas Chromatograph Clarus 680 and Mass Spectrometer Clarus SQ 8 C.

### Continuous-wave ESR

Experiments were performed on a home built quasi-optical spectrometer operated in the 55–420 GHz frequency range in a corresponding 0–16 T field range^43^,^44^. At low frequencies of 4, 9.4 and 34 GHz, a Bruker elexsys E500 spectrometer was used. For a typical experiment, about 1 mg of the carbon sample was weighed and then sealed in a quartz ESR tube after being heated at 500 K under dynamic vacuum overnight. For temperature- and frequency-dependent experiments, the magnetic field modulation amplitude was smaller than 0.01 mT and the microwave power was set to 0.2 μW to avoid signal distortion. For *g*-factor reference, a polycrystalline KC_60_ powder was used with *g*=2.0006.

### Pulsed ESR

Experiments were performed at 9.4 and 34 GHz using Bruker ElexSys 580 and 680 spectrometers. For *T*_2_ determination we used a simple 2-pulse sequence, invoking a first *π*/2 pulse of 16 ns, and an initial delay of the second pulse of 300 ns. The resulting echo was integrated over 175 ns. For *T*_1_, a 3-pulse sequence was used. An initial *π* pulse of 32 ns was followed after 300 ns delay by a 2-pulse echo sequence with 200 ns initial pulse delay for monitoring the inversion recovery. For Rabi experiments, a single π/2 pulse was used. Its length was incremented by 2 or 4 ns. After a delay of ∼84 ns with respect to the pulse ending, the signal was observed with a short integrating time of 16 ns.

### Data availability

The data that support the findings of this study are available from the corresponding authors upon request.

## Additional information

**How to cite this article:** Náfrádi, B. *et al*. Room temperature manipulation of long lifetime spins in metallic-like carbon nanospheres. *Nat. Commun.* 7:12232 doi: 10.1038/ncomms12232 (2016).

## Supplementary Material

Supplementary Information Supplementary Figures 1-12, Supplementary Table 1, Supplementary Notes 1-4 and Supplementary References

## Figures and Tables

**Figure 1:**
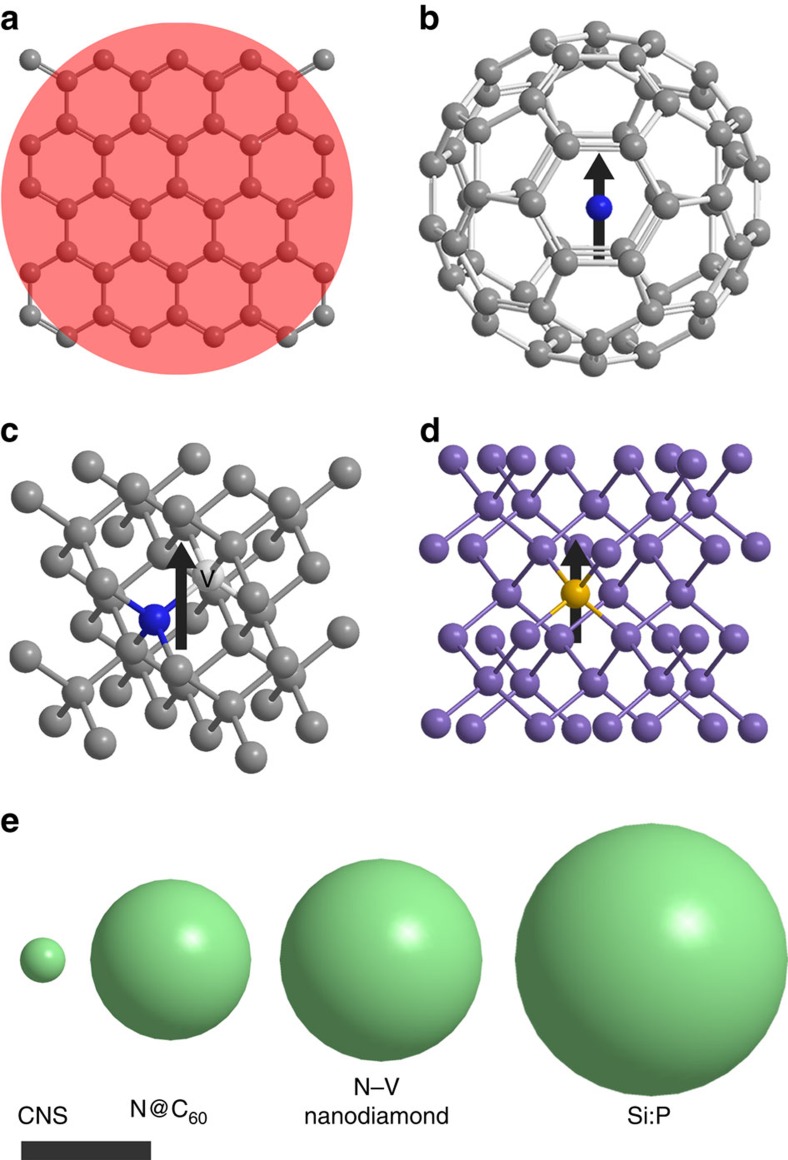
Comparison of itinerant and localized electron-based qubits. (**a**) Itinerant spin system of carbon nanospheres (CNS), and localized spin systems of (**b**) N@C_60_, (**c**) N-V nanodiamond and (**d**) Si:P. The spin information in the CNS is encoded by a delocalized electron spin that spreads over the entire 35-nm diameter (shaded area), making the system more robust against external magnetic field fluctuations and hyperfine interactions enforced by nuclear spins. Consequently, high qubit density can be achieved without enhanced decoherence. (**e**) The sphere diameters comparing the required volume for different types of qubits^14^,^25^,^50^ with scale bar, 100 nm.
