# Supplementary material for: Room temperature manipulation of long lifetime spins in metallic-like carbon nanospheres
Source: Nat Commun. 2016 Jul 18;7:12232. doi: 10.1038/ncomms12232 (PMC4960311; doi:10.1038/ncomms12232)
Supplement: Supplementary Information — Supplementary Figures 1-12, Supplementary Table 1, Supplementary Notes 1-4 and Supplementary References [file ncomms12232-s1.pdf]

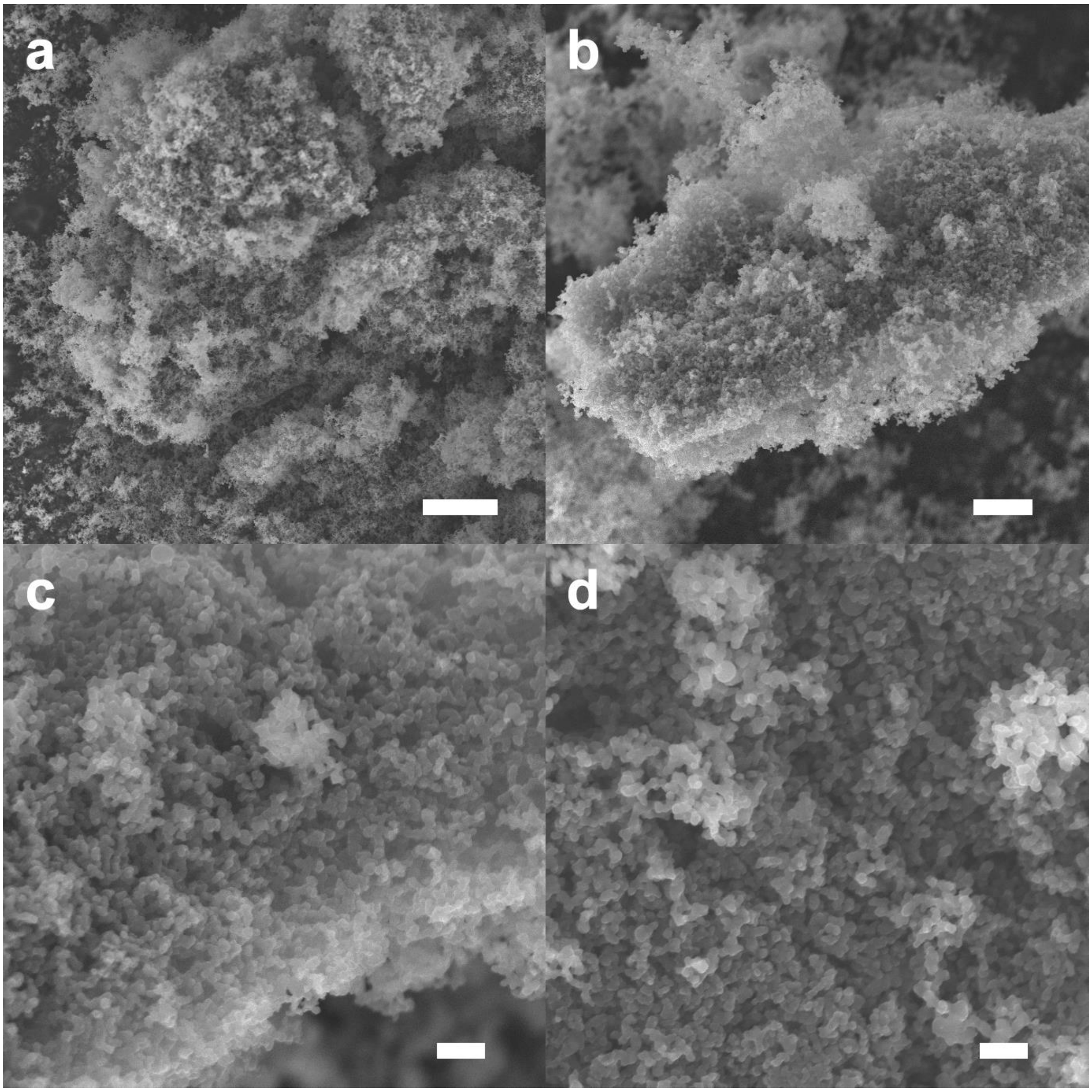

Supplementary Figure 1. SEM of the CNSs. (a) and (b) of the CNSs at low magnification, and (c) and (d) at higher magnification, with (c) being from a region in (b). Scale bars represent in (a) 5 µm, (b) 1 µm, (c) 200 nm, and (d) 200 nm. SEM images show an extensive formation of carbon nanoparticles spanning micron scales.

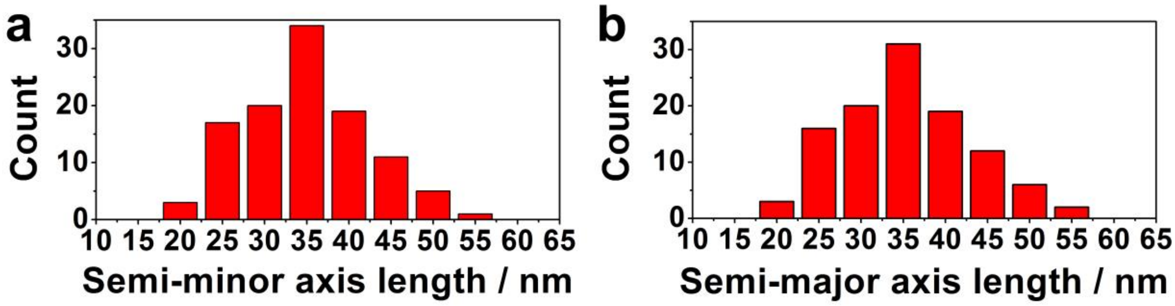

Supplementary Figure 2. Carbon nanosphere particle size distribution. (a) and (b) Particle size distribution histograms (35 nm range) obtained from the TEM image in Figure 2a assuming ellipsoidal particles with semi-major and semi-minor axis lengths plotted respectively.

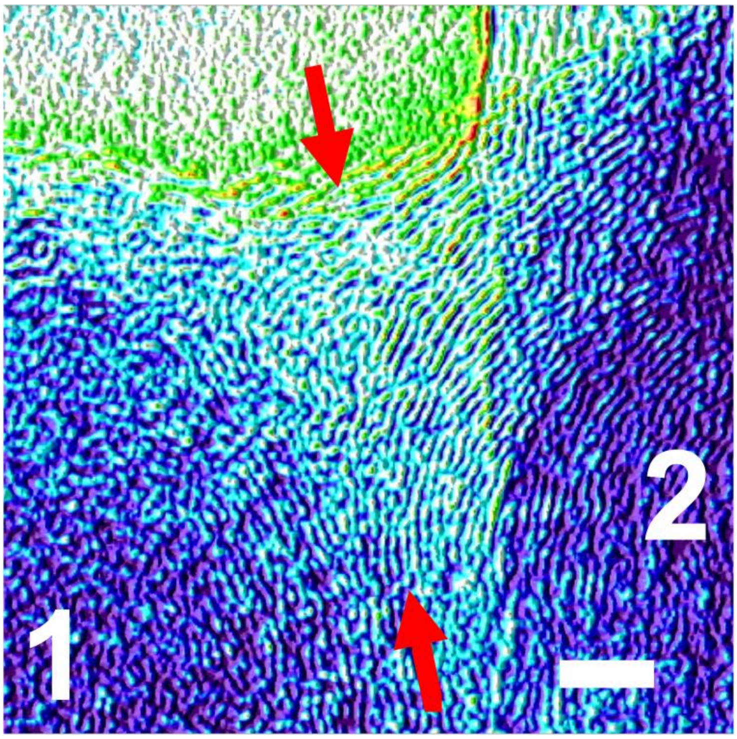

Supplementary Figure 3. Carbon nanosphere accretion. A high resolution TEM of two nanospheres (1 and 2) shown as a colour-surface topography with darker regions indicating shell overlap and the region of coalescence between them indicated by the arrows. Sphere 1 is suspended over a vacuum while 2 is on a carbon support, with ca. 15 outer layers coalescent. Scale bar represents 2 nm. The disordered non-crystalline carbon structure was observed to be maintained during heating.

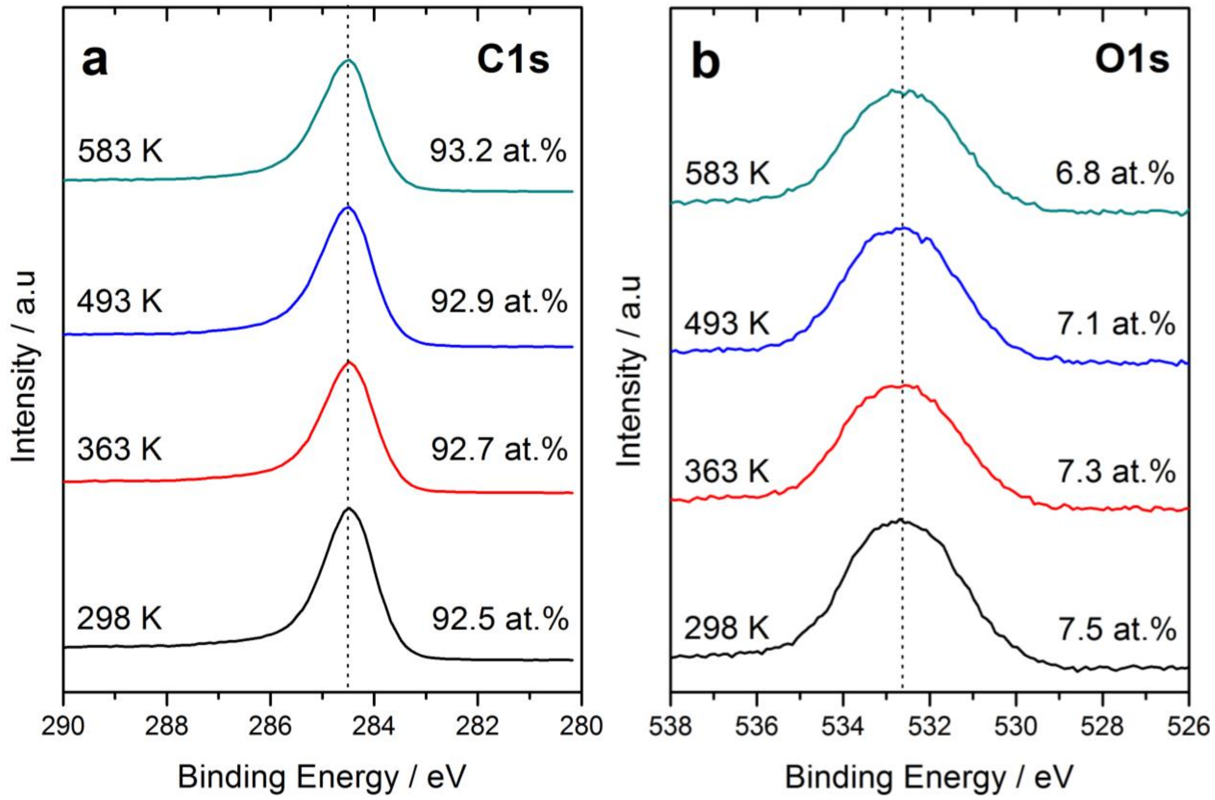

Supplementary Figure 4. XPS spectra of CNSs. The corresponding atomic percentage contributions of the core C 1*s* and O 1*s* lines are shown during in-situ heating. Dashed line in (a) is at 284.5 eV and in (b) 532.6 eV.

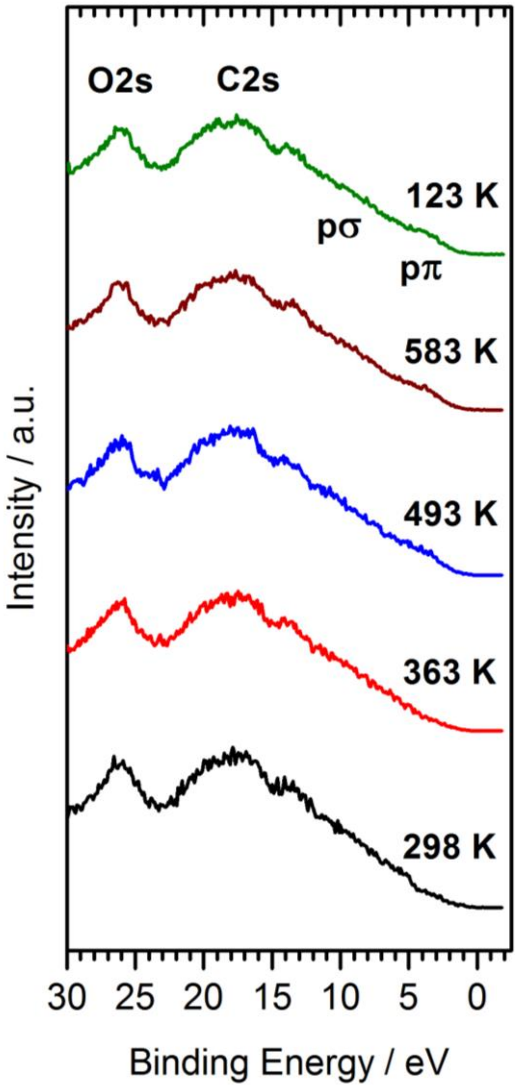

Supplementary Figure 5. *In-situ* valence band XPS spectra of CNSs at various temperatures. The material is first heated to 593 K then cooled to 123 K.

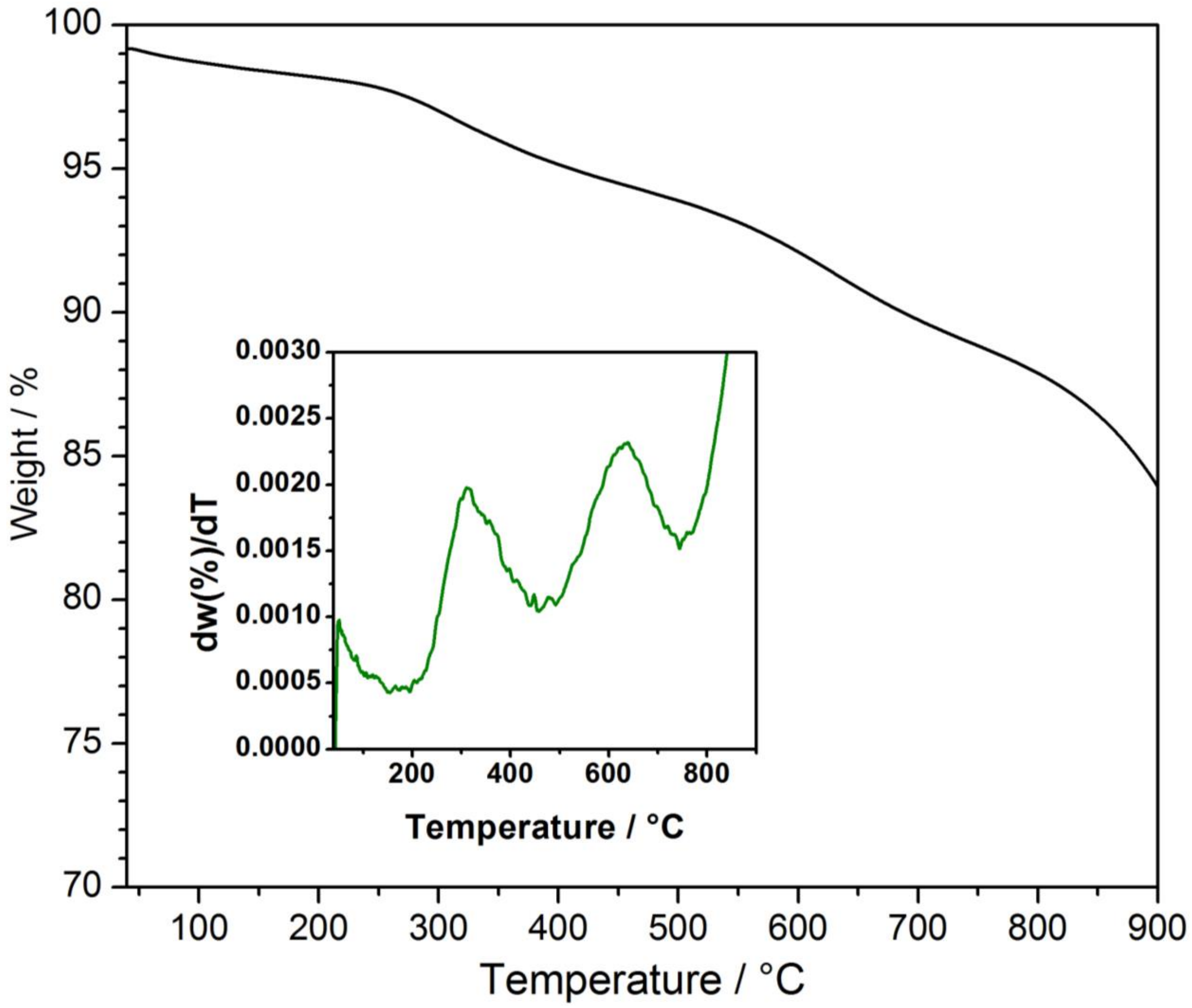

Supplementary Figure 6. High resolution TGA of the CNSs material. Inset shows the derivative of weight loss as a function of temperature with 2 prominent weight loss features at *ca.* 310°C and 610°C. Less than 2% of weight loss event occurred which can be attributed to degas and solvent losses (temperature up to 150°C).

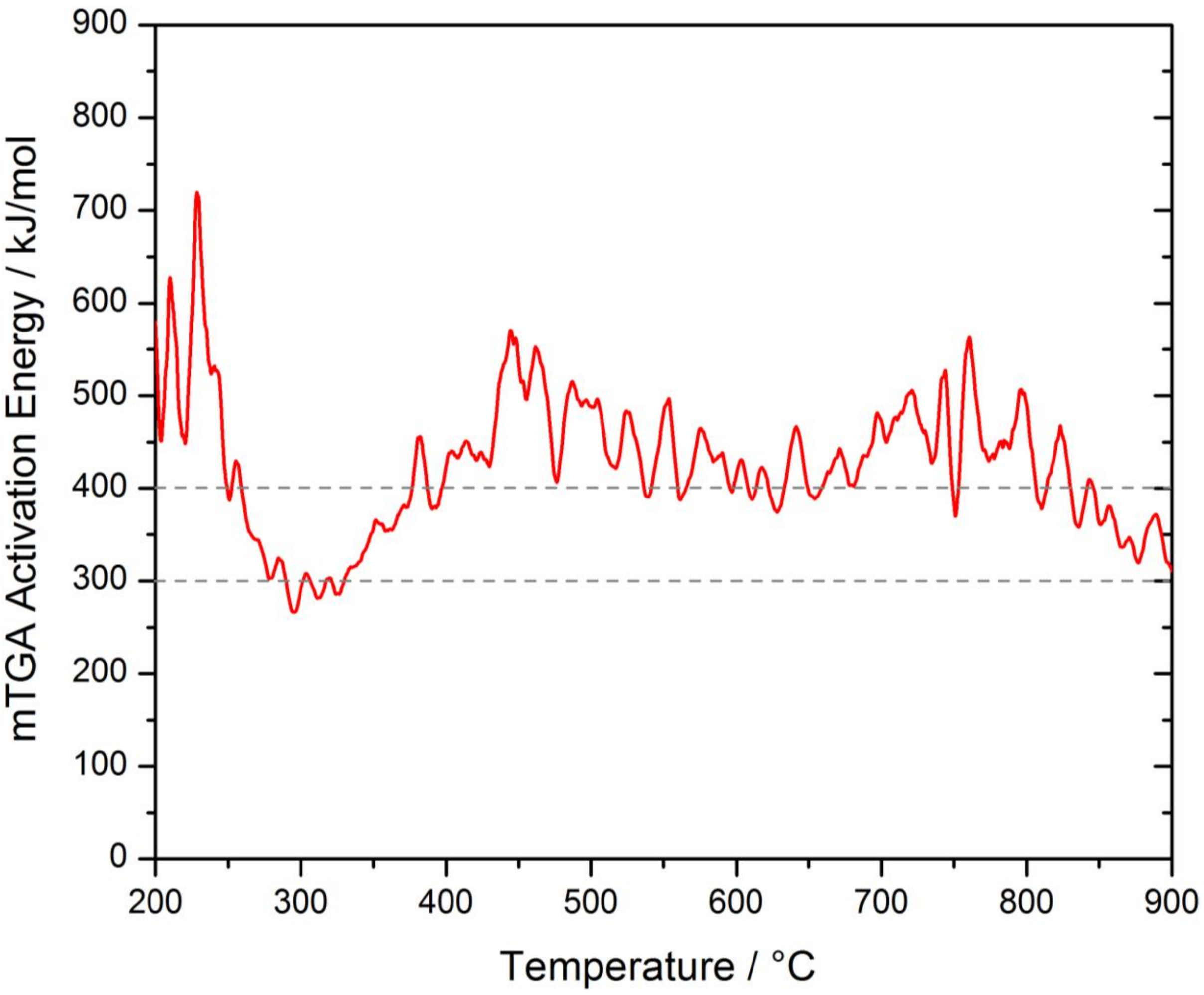

Supplementary Figure 7. Activation energy during heating to 900°C obtained from the modulated TGA experiment on the CNSs. The corresponding major weight loss events at *ca.* 310°C and 610°C have activation energies of *ca.* 300 kJ/mol and 400 kJ/mol respectively, indicating very slow kinetics of decomposition.

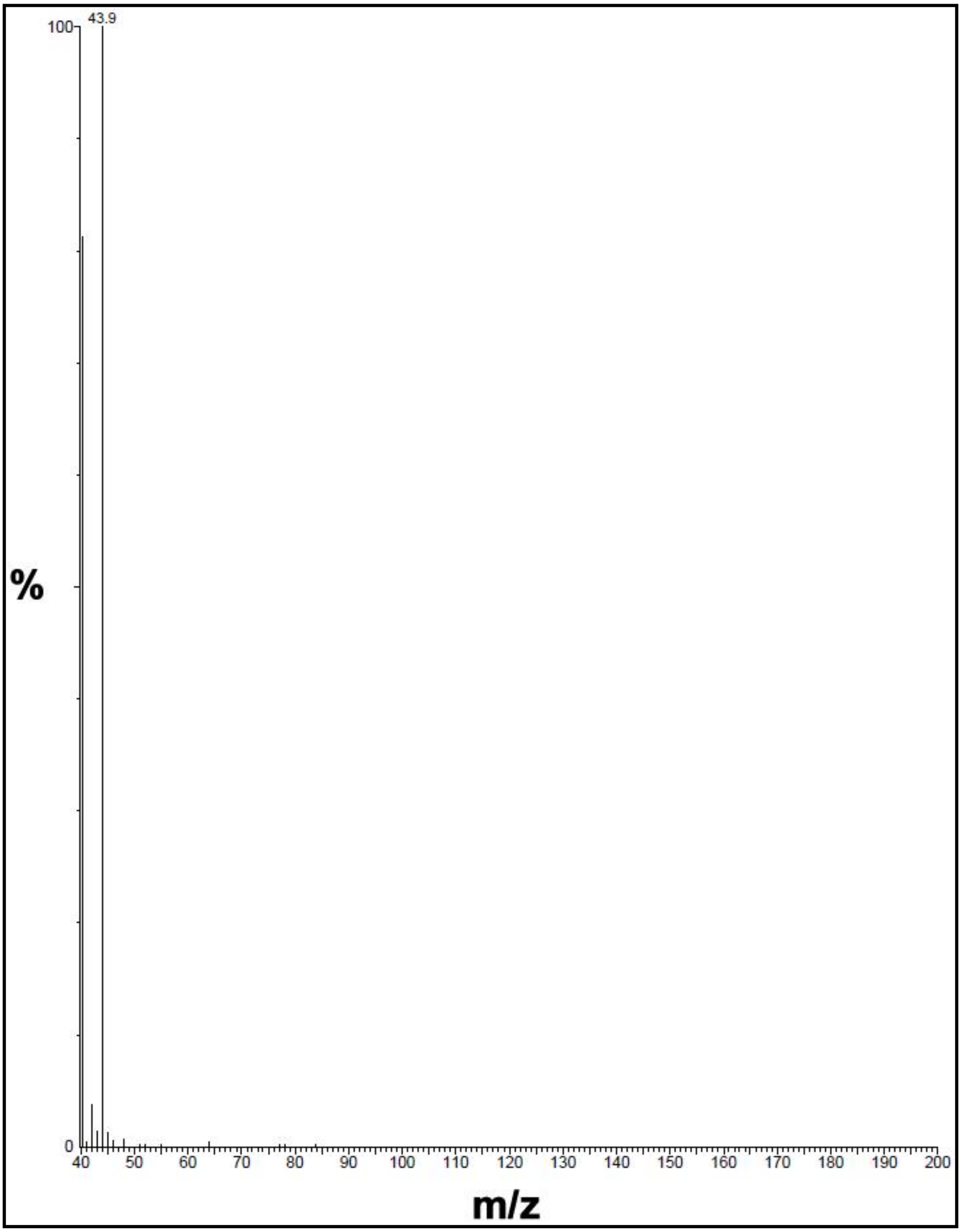

Supplementary Figure 8. Typical TGA-mass spectra. Heating of CNSs from 150°C to 610°C, showing a *m*/*z* corresponding to CO_2. No evidence of naphthalene or other polyaromatic hydrocarbons in the sample.

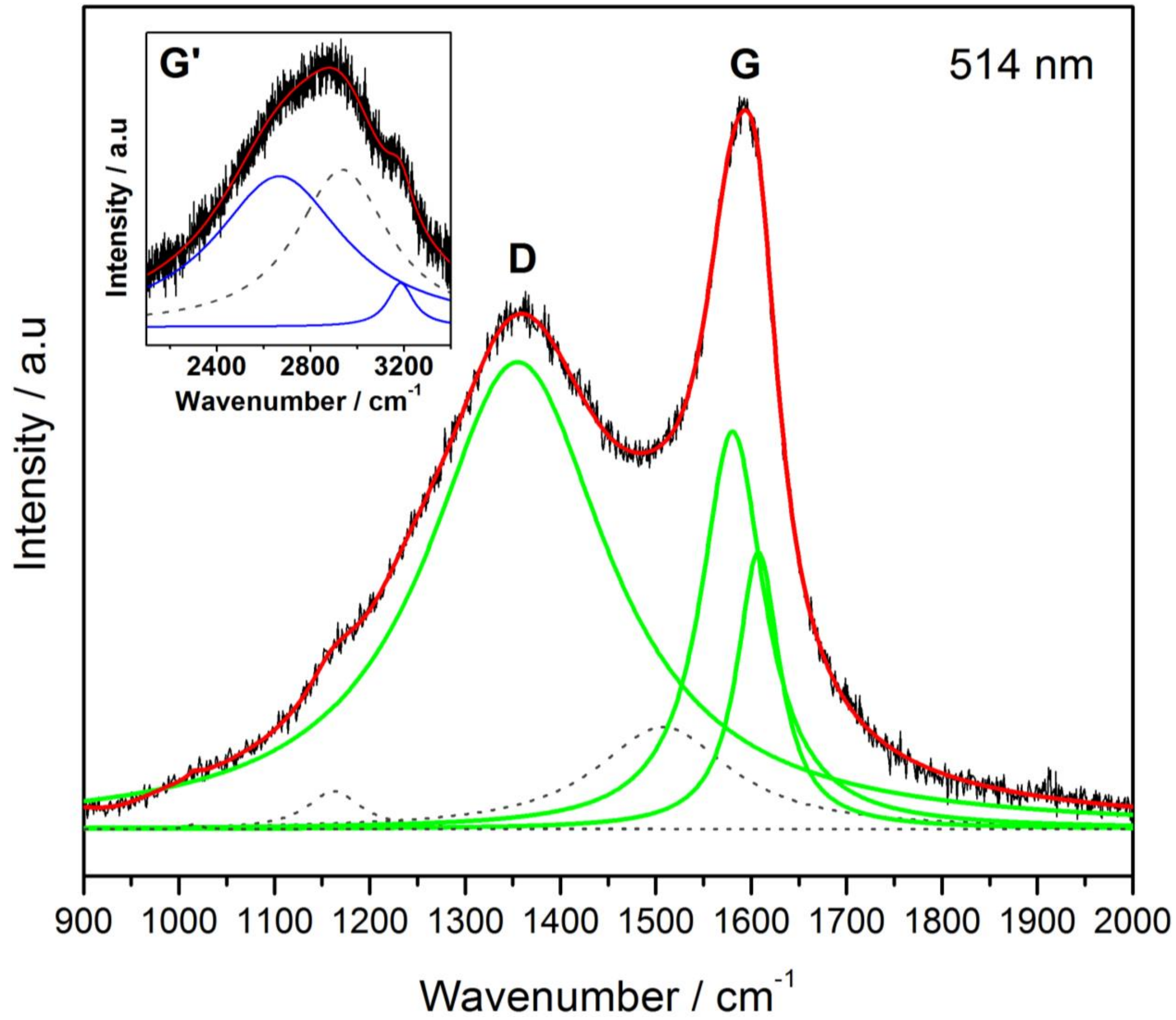

Supplementary Figure 9. Raman spectra of CNSs sample. Lorentzian line-shape peak fitting shown, with dashed lines representing band contributions from ethanol used to disperse the sample. Red outline is the envelope of the peak fitting. Second order peaks, G', shown in the inset.

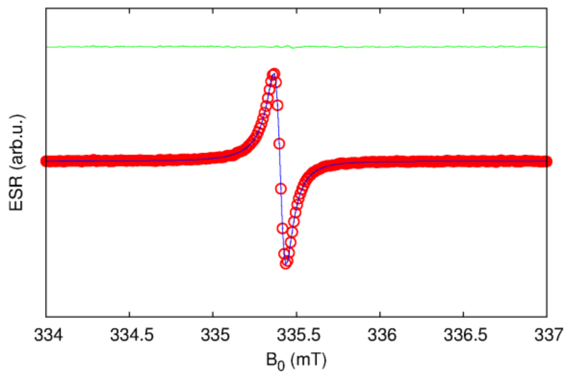

Supplementary Figure 10. Room temperature (300 K) ESR signal from the CNSs testifying the long conduction T_1=T_2. A fit to a derivative Lorentzian line-shape (blue line) and the near-zero residual signal (green line) indicating an excellent homogeneous line shape characteristic to itinerant electrons.

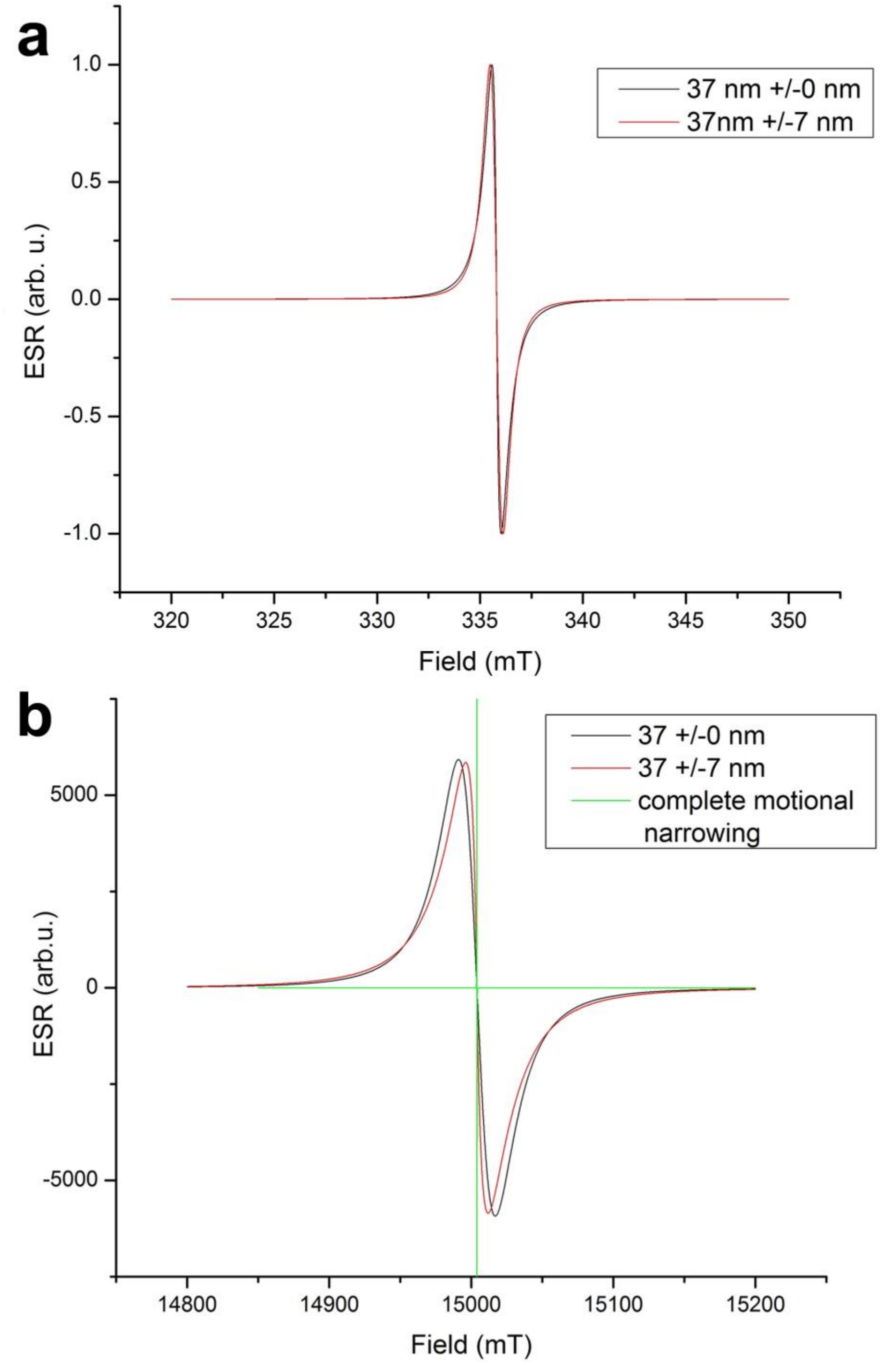

Supplementary Figure 11. (a) Simulated 9.4 GHz ESR spectra of carbon nanospheres based on eq. 1 and 2 with and without the contribution of size distribution, black and red lines respectively. The two spectra are practically identical due to the narrow size distribution. **(b)** Simulated 420 GHz ESR spectra of CNSs based on eq. 1 and 2 with and without the contribution of size distribution, black and red lines respectively. Green line is a simulated spectra assuming (unphysical) complete motional narrowing.

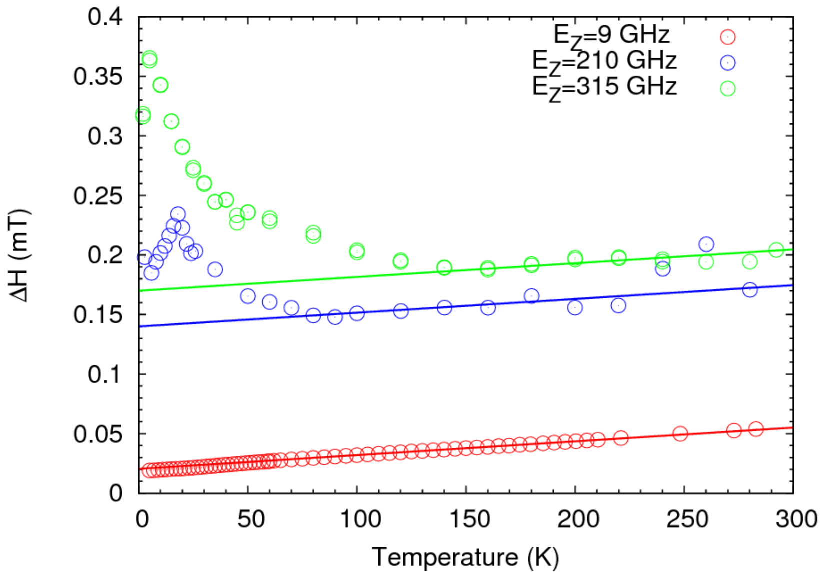

Supplementary Figure 12. Temperature dependence of the ESR linewidth measured at different strength of the Zeeman energy. At high-temperatures where no saturation effect occurs the linewidth decreases linearly by decreasing temperature. This behaviour is commonly observed in metals and explained by spin-orbit coupling by Elliott[12]. The slope is independent of E_Z at high temperatures also in agreement with Elliott mechanism.[12] The deviation from the linear dependence at high-frequencies and at low temperatures is due to ESR saturation effects which occurs progressively at higher temperatures by increasing E_Z.[13]

Supplementary Table 1. ***sp*^{2} to *sp*^{3} carbon content in the sample obtained from the XPS C 1*s* core line.**

**Temperature / K**	***sp*^{2} / %**	***sp*^{3} / %**
298	66	34
363	62	38
493	60	40
583	60	40

Supplementary Note 1 – X-ray Photoelectron Spectroscopy

The main core C 1*s* envelope was representative of an asymmetric peak commonly obtained for conducting graphitic materials; having a low level of oxidation and a very narrow peak width at half-maximum (FHWM less than 1.2 eV) and positioned at a binding energy corresponding to pure graphitic material 284.5 eV (Supplementary Figure 4a)[2,3]. Upon heating, the C 1*s* peak did not shift from the *sp*^{2} graphite binding energy position of 284.5 eV or change notably in width at half maximum.

The total atomic ratio at 298 K of carbon to oxygen was *ca.* 12:1 which increased to *ca.* 14:1 as the material was heated, Figure S4. The increase in temperature resulted in the removal of oxygen in the form of CO_2 (Supplementary Figure 8). Curve fitting employed for the O 1*s* line indicated both O=C (533.2 eV) and O–C (531.8 eV) chemical bonding environments were present in the onion-like carbon nanospheres. It was inappropriate to quantify the individual oxygen environments present as there was low total oxygen content (less than 10 at.%), a small difference in oxygen lost during heating (less than 0.8 at.%), and a broad O 1*s* line peak (FHWM *ca.* 3.8 eV), Supplementary Figure 4b. The XPS results indicated that the CNSs remained chemically and thermally stable even up to temperatures of 583 K.

The valence band XPS spectrum (Supplementary Figure 5) shows a fairly broad, intense peak located between 16 and 23 eV, a narrower less intense peak with a well-defined minimum located between 12 to 15 eV (both assigned to C 2*s*), and a very broad and decidedly weaker structure tailing off and extending from 12 eV to the cut-off energy (*p*-σ peak) typical of graphitic material.[4-6] The C 2*s* peak had two peaks (10–25 eV) which strongly suggested the presence of an sp^2 network made up of six-fold rings, as this feature is known to 'wash-out' by the presence of an increased number odd-membered rings in a random network.[5] A single O 2*s* contribution is also observed between 24 to 29 eV.[6] The positions of the band peaks do not change with temperature. The *p*-π states are not apparent in the as prepared CNSs and only appear as a shoulder on the leading edge of *p*-σ peak after annealing beyond 363 K and persists even when cooling to 123 K. The emergence of the *p*-π band may arise from changes in the p_z wave functions at large radii due to the delocalised nature of the *p*-π orbitals.[4] The evolution of *p*-π states with temperature closely resembled that observed for amorphous/non-crystalline carbon (above 623 K in amorphous carbon)[5], and the presence of an O 2*s* contribution was similar to that observed in partially oxidised graphitic fibres[6].

Supplementary Note 2– Thermo-gravimetric Analysis

In Supplementary Figure 6 two weight loss events occurred very distinctly, at 583 K and 883 K, attributed to the removal of chemically bound oxygen in the form of CO_2, (see Supplementary Figures 4 and 7). Less than 2 weight percent (wt.%) was lost at 493 K (attributed to degassing, removal of adsorbed H_2O), less than 5 wt.% at 583 K, and remarkably only less than 10 wt.% at 923 K. The large activation energies associated with the main weight loss events (300–400 kJ/mol) were evidence of very slow decomposition of the carbon, Figure S6. However, it remains unclear whether the removal of oxygen from O–C and O=C groups occurs separately.

Supplementary Note 3 – Raman Spectroscopy

The Raman spectra of the carbon material best represents a disordered carbon material between nano-crystalline graphite to low sp^2 non-crystalline carbon, Figure S9[7], which agreed well with XPS analysis on the ratio of sp^2 to sp^3 carbon (Supplementary Table 1). Lorentzian curve fitting employed on the Raman spectrum of the graphene material showed an asymmetry in the 'G band' yielding peaks centered at 1580 cm^{-1} with FWHM 80 cm^{-1} and 1607 cm^{-1} with FHWM 54 cm^{-1} with decreasing relative intensity. This G 'band' is believed

to be due to the in-plane stretching motion between pairs of sp^2 carbon atoms. This mode does not require the presence of six-fold rings, so it occurs at all sp^2 sites not only those in rings, and appears in the range 1500–1630 cm^{-1}. The asymmetry in the peak may be caused by doping of the graphitic layers by ethanol present which was used to disperse the sample prior to measurement[8].

The presence of the 'D band' centered at 1355 cm^{-1} with FWHM 230 cm^{-1}, is believed to be related to the number of ordered aromatic rings, and affected by the probability of finding a six-fold ring in a cluster. The second order peaks (G') are not well defined, but appear as a small modulated bump between 2200 and 3500 cm^{-1} and best represent a multi-layer graphitic material in the presence of some ethanol.

The intensity ratio of the D band to the G band(s) value, commonly reported as I_D/I_G, was 1.2 and 1.7, indicating a significant number of defect sites present.[7,9] This value compares well with reported I_D/I_G values for carbon nanospheres, which range between 0.8–1.2.[10] The relative intensity and positions of the G and D bands have been interpreted to be due to the presence of defects and disorder in the short range graphitic fragments.[11] This is directly verified with TEM (Figure 3 and Supplementary Figure 3). The identification of bands associated with other phases, which may also be present in smaller quantities (*e.g.* diamond), was not possible due to the background of the Raman spectrum contributions of disordered carbon.

Supplementary Note 4 – Electron Spin Resonance

ESR experiments revealed a presence of a single narrow (ΔH=0.05 mT) Lorentzian line with g=2.00225 at 9.4 GHz frequency (Supplementary Figure 10). The spectral resolution of ESR is proportional to the frequency. The deviations from the Lorentzian shape even at 420 GHz were smaller than 5%. There was no g-factor anisotropy observed within the resolution of the 420 GHz measurements of $\Delta g<10^{-6}$. Note that in carbon the g-factor values of localized paramagnetic centres are in the 2.0025-2.0050 range with anisotropies Δg in the order of $\sim 5\times 10^{-4}$.[1]

A spin-½ system in magnetic field $\mathbf{B_0}$ is a two-level quantum system which can be a physical representation of a qubit. If an oscillating magnetic field is applied in such that the total magnetic field $\mathbf{B}$ acting on the spin is $\mathbf{B}=B_0\mathbf{z}+B_1(\sin(\omega t)\mathbf{x}+\cos(\omega t)\mathbf{y})$ the qubit will oscillate between the states |+½> and |-½>. Let the q-bit be in state |-½> at t=0. The probability to find the q-bit in state |+½> at time t is

$$P(t) = (\omega_1/\mathbf{\Omega})^2\sin^2(\Omega t/2)$$

where $\mathbf{\Omega} = \sqrt{(\omega-\omega_0)^2 + \omega_1^2}$; and $\omega_0 = \gamma B_0$, $\omega_1 = \gamma B_1$, and γ is the gyromagnetic ratio.

This is called Rabi oscillation. Thus the detected Rabi oscillations of several cycles can be taken as evidence that the system allows the deliberate preparation of any superposition of a two level spin-½ system. For example, to go from one state |+½> to |-½> we can adjust the time t during which the oscillating field acts such that $\omega_1 t/2 = \pi/2$ (*i.e.* $t = \pi/\omega_1$); this is called

a π pulse. If a time intermediate between 0 and π/ω_1 is chosen, *e.g.* in the case for $t = \pi/2\omega_1$, we obtain a $\pi/2$ pulse, and this results in a superposition of $\sqrt{2}*(|+½> + |-½>)$ of the two states.

For conduction electron spin based qubits the size distribution on the ESR relaxation rate has little effect. This is in contrasting difference from localized paramagnetic spin based q-bits like N@C_{60} N-V centers or other molecular magnet based systems. The ESR relaxation at E_Z=0 is limited by T_1 because the motional narrowing of conduction electrons is complete. Thus the ESR line is homogeneous and independent of the size distribution. Inhomogeneous broadening comes about at high magnetic fields. The complete motional narrowing of conduction electrons breaks down as electrons progressively confine to cyclotron orbits. This gives the linear broadening by field described by Equation 1 of the manuscript. The finite size distribution of the particles induces additional inhomogeneity because the slope of the field dependent broadening depends on the particle size (Equation 2 of the manuscript). At the typical ESR frequency at X-band (Supplementary Figure 11a) the size distribution induced broadening is negligible. At high frequencies the broadening induced by size distribution is enhanced, however, it is still negligible compared to the magnetic field induced broadening as shown in Supplementary Figure 11b.

Supplementary References

1 Iakoubovskii, K., Stesmans, A., Suzuki, K., Kuwabara, J. & Sawabe, A. Characterization of defects in monocrystalline CVD diamond films by electron spin resonance. *Diamond and Related Materials* **12**, 511-515 (2003).

2 Estrade-Szwarckopf, H. & Rousseau, B. Photoelectron core level spectroscopy study of Cs-graphite intercalation compounds—I. Clean surfaces study. *Journal of Physics and Chemistry of Solids* **53**, 419-436 (1992).

3 Smith, K. L. & Black, K. M. Characterization of the treated surfaces of silicon alloyed pyrolytic carbon and SiC. *Journal of Vacuum Science & Technology A* **2**, 744-747, (1984).

4 McFeely, F. R. *et al.* X-ray photoemission studies of diamond, graphite, and glassy carbon valence bands. *Physical Review B* **9**, 5268-5278 (1974).

5 Robertson, J. Amorphous carbon. *Advances in Physics* **35**, 317-374, doi:10.1080/00018738600101911 (1986).

6 Xie, Y. & Sherwood, P. M. A. X-ray photoelectron-spectroscopic studies of carbon fiber surfaces. 11. Differences in the surface chemistry and bulk structure of different carbon fibers based on poly(acrylonitrile) and pitch and comparison with various graphite samples. *Chemistry of Materials* **2**, 293-299, doi:10.1021/cm00009a020 (1990).

7 Ferrari, A. C. & Robertson, J. Interpretation of Raman spectra of disordered and amorphous carbon. *Physical Review B* **61**, 14095-14107 (2000).

8 Humberto, T., Ruitao, L., Mauricio, T. & Mildred, S. D. The role of defects and doping in 2D graphene sheets and 1D nanoribbons. *Reports on Progress in Physics* **75**, 062501 (2012).

9 Ferrari, A. C. & Basko, D. M. Raman spectroscopy as a versatile tool for studying the properties of graphene. *Nat Nano* **8**, 235-246 (2013).

10 Nieto-Marquez, A., Romero, R., Romero, A. & Valverde, J. L. Carbon nanospheres: synthesis, physicochemical properties and applications. *Journal of Materials Chemistry* **21**, 1664-1672, doi:10.1039/c0jm01350a (2011).

11 Obraztsova, E. D. *et al.* Raman identification of onion-like carbon. *Carbon* **36**, 821-826 (1998).

12 Elliott, R. J. Theory of the Effect of Spin-Orbit Coupling on Magnetic Resonance in Some Semiconductors. *Physical Review* **96**, 266-279 (1954).

13 Poole Jr, C. P. & Farach, H. A. in *Relaxation in Magnetic Resonance* (eds Charles P. Poole & Horacio A. Farach) 17-29 (Academic Press, 1971).
